# Supplementary material for: Rapid On-Farm Testing of Resistance in Lolium rigidum to Key Pre- and Post-Emergence Herbicides
Source: Plants (Basel). 2021 Sep 10;10(9):1879. doi: 10.3390/plants10091879 (PMC8467281; doi:10.3390/plants10091879)
Supplement: Supplementary file 1 [file plants-10-01879-s001.zip › plants-1355769-supplementary.pdf]

Table S1. Dormancy-breaking treatments tested on dormant and conditionally-dormant seeds.

Treatment type	Treatment[1]	Post-treatment	Reference[2]
Acid scarification	Conc. H_2SO_4 x 5 min	Rinse 5 min in running water	[1]
Acid scarification	Conc. HCl x 5 min	As above	[3]
Acid scarification	5.5 M HCl x 10 min	As above	[3]
Acid scarification	Conc. H_2SO_4 x 5 min	Rinse, then 1.5% KNO_3 x 2 h	[1]
Alkaline scarification	35% NaOH x 10 min	Rinse 5 min in running water	[3]
Heat	80°C water x 10 min	Rinse briefly in cold water	[1]
Reactive oxygen species	3% H_2O_2 x 6 h	Rinse 3 min in running water	[5]
Reactive oxygen species	Fenton reagent (1% H_2O_2 + 3 mg mL^{-1} $FeSO_4$) x 6 h	As above	[5]
Reactive oxygen species	0.1 mM methyl viologen x 3 h	As above	[5]
Mechanical scarification	Cut through edge of endosperm (dry seeds)	N/A	-
Mechanical scarification	Cut through edge of endosperm (24-h imbibed seeds)	N/A	-
Mechanical scarification	Rub dry seeds with sandpaper	N/A	[3]
Chemical stimulant	Imbibe in 1% KNO_3, 10 μM GA_4 or 50 μM fluridone x 24 h	N/A	[4]
Chemical stimulant	Imbibe in 1% KNO_3 + 10 μM GA_4 x 24 h	N/A	[4]
Chemical stimulant	Sow on agar containing 1% KNO_3, 10 μM GA_4 or 50 μM fluridone	N/A	[4]
Chemical stimulant	Sow on agar containing 1% KNO_3 + 10 μM GA_4	N/A	[4]
Chemical stimulant	Sow on agar containing 10 μM GA_4 + 50 μM fluridone	N/A	[4]
Chemical stimulant	Sow on agar containing 1 – 100 μM AA1	N/A	[6]
Chemical stimulant	Sow on agar containing 2 μM AA1 plus 10 μM GA_4 and/or 50 μM fluridone	N/A	[4,6]
Chemical stimulant	Incubate seeds in 250 mL aerated water x 24 h (dark) to leach ABA	Allow seeds to dry, then sow on agar containing 10 μM GA_4	[2]
Chemical stimulant	Leach as above, then incubate in 500 μM AA1 x 2 h	As above	[2,6]

[1]Abbreviations/chemical symbols: AA1: abscisic acid antagonist 1; ABA: abscisic acid; $FeSO_4$: iron (II) sulphate; GA_4: gibberellin A_4; HCl: hydrochloric acid; H_2O_2: hydrogen peroxide; H_2SO_4: sulphuric acid; KNO_3: potassium nitrate; NaOH: sodium hydroxide.

[2]References: [1] Baličević, R.; Ravlić, M.; Balić, A. *J. Cent. Eur. Agric*. **2016**, *17*, 725–733; [2] Braun, J.W.; Khan, A.A. *Plant Physiol.* **1975** *56*, 731–773; [3] Burton, G.W. *J. Am. Soc. Agron.* **1939**, *31*, 179–187; [4] Goggin, D.E.; Emery, R.J.N.; Powles, S.B.; Steadman, K.J. *J. Plant Physiol.* **2010**, *167*, 1282–1288; [5] Whitaker, C.; Beckett, R.P.; Minibayeva, F.V.; Kranner, I. *S Afr J Bot* **2010**, *76*, 601–605; [6] Ye, Y.; Zhou, L.; Liu, X.; Liu, H.; Li, D.; Cao, M.; Chen, H.; Xu, L.; Zhu, J-K.; Zhao, Y. *Plant Physiol* **2017**, *173*, 2356–2369.

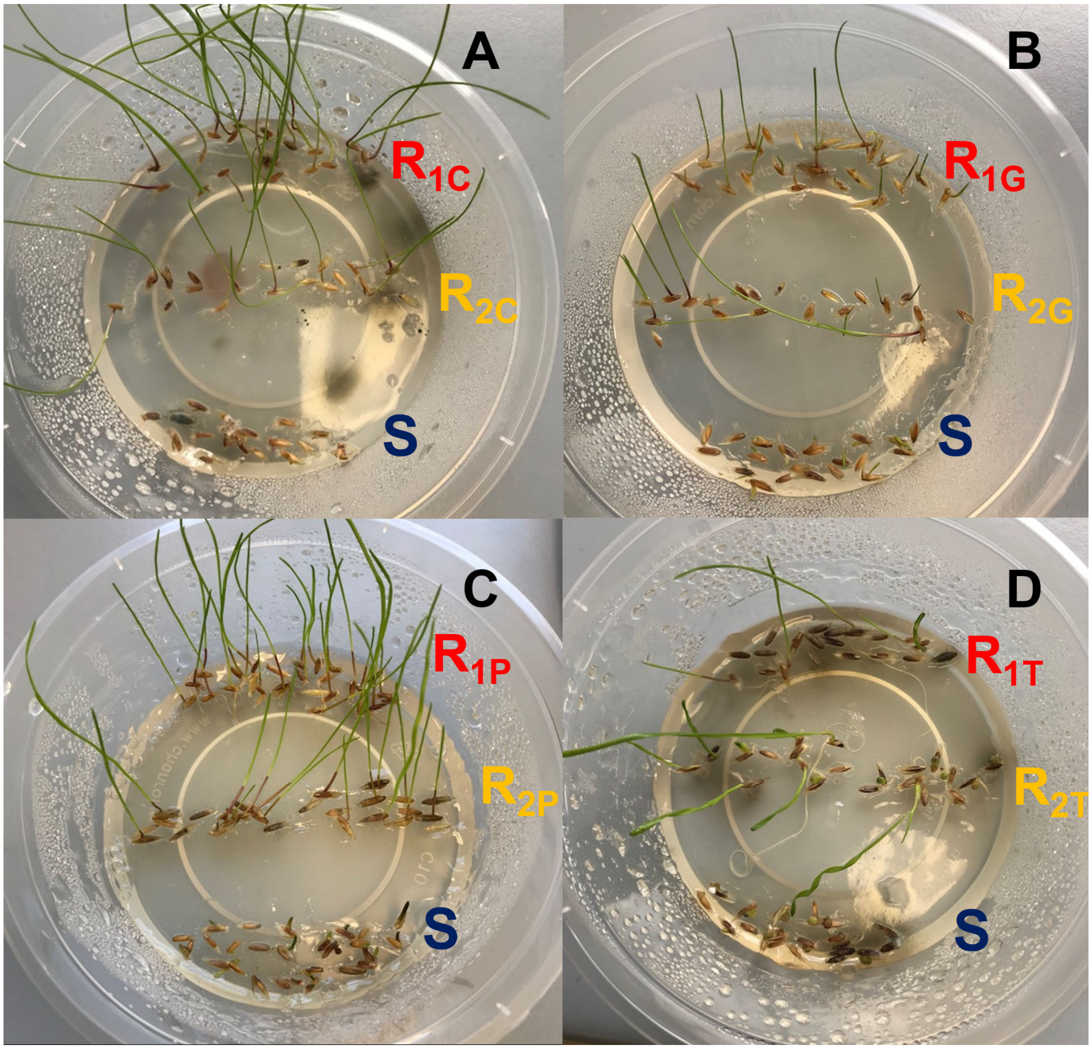

Figure S1. Difference in response between two herbicide-resistant (R_1, R_2) and one herbicide-susceptible (S) population to: A) clethodim (1 µM), B) glyphosate (400 µM), C) pyroxasulfone (0.15 µM) and D) trifluralin (25 µM); the seeds of R populations germinate and develop a 3–5 cm coleoptile (and roots) after 7 d of incubation on herbicide-impregnated agar, whereas the seed from the S population germinates but fails to develop.
